# Patterns in Outpatient Benzodiazepine Prescribing in the United States

**DOI:** 10.1001/jamanetworkopen.2018.7399

**Published:** 2019-01-25

**Authors:** Sumit D. Agarwal, Bruce E. Landon

**Affiliations:** 1 Division of General Internal Medicine and Primary Care, Department of Medicine, Brigham and Women’s Hospital, Boston, Massachusetts; 2 Harvard Medical School, Boston, Massachusetts; 3 Department of Health Care Policy, Harvard Medical School, Boston, Massachusetts; 4 Division of General Medicine and Primary Care, Department of Medicine, Beth Israel Deaconess Medical Center, Boston, Massachusetts

## Abstract

**Importance:**

Benzodiazepines are implicated in a growing number of overdose-related deaths.

**Objectives:**

To quantify patterns in outpatient benzodiazepine prescribing and to compare them across specialties and indications.

**Design, Setting, and Participants:**

This serial cross-sectional study (January 1, 2003, through December 31, 2015) used nationally representative National Ambulatory Medical Care Survey data. The yearly population-based sample of outpatient visits among adults, ranging from 20 884 visits in 2003 (representing 737 million visits) to 24 273 visits in 2015 (representing 841 million visits) was analyzed. Prescribing patterns were examined by specialty and indication and used to calculate the annual coprescribing rate of benzodiazepines with other sedating medications. Data were analyzed from July 1, 2017, through November 30, 2018.

**Main Outcomes and Measures:**

Annual benzodiazepine visit rate.

**Results:**

Among the 386 457 ambulatory care visits from 2003 through 2015, a total of 919 benzodiazepine visits occurred in 2003 and 1672 in 2015, nationally representing 27.6 million and 62.6 million visits, respectively. The benzodiazepine visit rate doubled from 3.8% (95% CI, 3.2%-4.4%) to 7.4% (95% CI, 6.4%-8.6%; *P* < .001) of visits. Visits to primary care physicians accounted for approximately half of all benzodiazepine visits (52.3% [95% CI, 50.0%-54.6%]). The benzodiazepine visit rate did not change among visits to psychiatrists (29.6% [95% CI, 23.3%-36.7%] in 2003 to 30.2% [95% CI, 25.6%-35.2%] in 2015; *P* = .90), but increased among all other physicians, including primary care physicians (3.6% [95% CI, 2.9%-4.4%] to 7.5% [95% CI, 6.0%-9.5%]; *P* < .001). The benzodiazepine visit rate increased slightly for anxiety and depression (26.6% [95% CI, 22.6%-31.0%] to 33.5% [95% CI, 28.8%-38.6%]; *P* = .003) and neurologic conditions (6.8% [95% CI, 4.8%-9.5%] to 8.7% [95% CI, 6.2%-12.1%]; *P* < .001), but more so for back and/or chronic pain (3.6% [95% CI, 2.6%-4.9%] to 8.5% [95% CI, 6.0%-11.9%]; *P* < .001) and other conditions (1.8% [95% CI, 1.4%-2.2%] to 4.4% [95% CI, 3.7%-5.2%]; *P* < .001); use did not change for insomnia (26.9% [95% CI, 19.3%-36.0%] to 25.6% [95% CI, 15.3%-39.6%]; *P* = .72). The coprescribing rate of benzodiazepines with opioids quadrupled from 0.5% (95% CI, 0.3%-0.7%) in 2003 to 2.0% (95% CI, 1.4%-2.7%) in 2015 (*P* < .001); the coprescribing rate with other sedating medications doubled from 0.7% (95% CI, 0.5%-0.9%) to 1.5% (95% CI, 1.1%-1.9%) (*P* < .001).

**Conclusions and Relevance:**

The outpatient use of benzodiazepines has increased substantially. In light of increasing rates of overdose deaths involving benzodiazepines, understanding and addressing prescribing patterns may help curb the growing use of benzodiazepines.

## Key Points

**Question:**

How are benzodiazepines being prescribed, and how have prescribing patterns changed over time?

**Findings:**

In this serial cross-sectional study of 386 457 ambulatory care visits from 2003 through 2015, the use of benzodiazepines in ambulatory care increased substantially from 3.8% to 7.4% of visits, including coprescribing with other sedating medications. Use among psychiatrists was stable (29.6% vs 30.2%) but increased among all other types of physicians, including primary care physicians (3.6% vs 7.5%), who as a group accounted for about half of all benzodiazepine visits.

**Meaning:**

In light of increasing death rates associated with benzodiazepine overdose, addressing prescribing patterns may help curb the growing use of benzodiazepines.

## Teaser

This cross-sectional study uses data from the National Ambulatory Medical Care Survey to quantify patterns in outpatient benzodiazepine prescribing and compare them across specialties and indications.

## Introduction

Benzodiazepine-related overdose mortality has risen dramatically, from 0.6 per 100 000 adults in 1999 to 4.4 per 100 000 in 2016.^1,2,3^ Benzodiazepines are also involved in many presentations to the emergency department as well as falls and fractures, motor vehicle crashes, and cognitive impairment.^4,5^ These risks are more pronounced when benzodiazepines are combined with alcohol, opioids, or other medications that affect the central nervous system (CNS).^6^ For these reasons, the Beers criteria recommend avoiding benzodiazepine prescribing among elderly patients.^7^

Although benzodiazepines are beginning to garner more attention amid the opioid crisis,^8^ recent focus has tended to be on the elderly,^9,10,11^ coprescribing with opioids,^12,13^ and patient factors that include white race, poor sleep quality, and certain comorbidities, such as lung disease and substance use disorder.^14,15^ Less is known about who prescribes benzodiazepines and for what indications or about coprescribing with other sedative medications beyond opioids. As a large class of medications with many potential indications—anxiety, panic, insomnia, seizures, alcohol withdrawal, muscle spasms, and neuropathic pain—additional information about prescribing patterns could help physicians and policy makers elucidate and address the alarming rise in benzodiazepine-related morbidity and mortality.

In this study, we used nationally representative data on outpatient visits in the United States to examine overall trends in benzodiazepine use in ambulatory care and to compare patterns across specialties and indications. Given the higher risks associated with taking multiple medications that affect the CNS, we also examined trends in coprescribing between benzodiazepines with opioids and other sedating medications.

## Methods

### Primary Data Source

We analyzed patient visits from the National Ambulatory Medical Care Survey (NAMCS) from January 1, 2003, through December 31, 2015. The NAMCS is an annual cross-sectional survey of ambulatory care visits in the United States, conducted by the National Center for Health Statistics. The NAMCS is nationally representative of outpatient visits to nonfederal, office-based physicians. The serial nature of the survey makes it ideally suited to track practice patterns over time. This study followed the Strengthening the Reporting of Observational Studies in Epidemiology (STROBE) reporting guideline. The Partners Human Research Committee deemed that this study was exempt from review and informed consent.

The NAMCS uses a multistage probability sample design. In the first stage, 112 geographically based primary sampling units are selected. In the second stage, practicing physicians, stratified by specialty, are selected within each sampling unit. Physicians are identified using master files maintained by the American Medical Association and American Osteopathic Association. In the third and final stage, patient visit data are collected from each selected physician during a randomly assigned 1-week reporting period.

For each sampled visit, standardized forms are used to collect data on patient demographic characteristics, chief complaints, diagnoses derived from the *International Classification of Diseases, Ninth Revision, Clinical Modification* (*ICD-9-CM*), and medications ordered, supplied, administered, or continued at the visit. From 2003 through 2015, the mean (SD) response rate among physicians was 53.5% (12.2%). Adjustments were applied using survey weights to minimize the effect of nonresponse bias.^16^ Item nonresponse rates were generally less than 5%, with the exception of race and insurance, which, depending on the year, carried a nonresponse rate to 33%; missing demographic data were imputed. Use of survey weights as outlined by the National Center for Health Statistics enables the calculation of national-level estimates and associated SEs. Additional details for the NAMCS can be found on the National Center for Health Statistics website.^17^

### Outcome Measures

From the NAMCS, we estimated the benzodiazepine visit rate among adults (aged ≥18 years) for each year during the 13-year study period. The denominator was the total number of visits; the numerator was the number of visits with a benzodiazepine prescription noted in the medical record.

From 2003 to 2011, as many as 8 medications could be recorded in the NAMCS. This number increased to 10 medications in 2012 and 2013 and subsequently to 40 medications in 2014 and 2015. To maintain consistency over time, we restricted our analyses to benzodiazepines that were coded within the first 8 medication positions, which eliminates in the most recent years about 5% to 15% of visits in which a benzodiazepine was noted and therefore slightly underestimates the overall benzodiazepine visit rate.

We identified all generic and brand-name benzodiazepines coded in the NAMCS, including alprazolam, chlordiazepoxide hydrochloride, clobazam, clonazepam, clorazepate dipotassium, diazepam, estazolam, flurazepam hydrochloride, lorazepam, midazolam hydrochloride, oxazepam, and temazepam. From 2005 (when the variable became available) to 2015, new prescriptions were distinguished from continuing prescriptions. We classified each benzodiazepine as short acting (half-life ≤24 hours) or long acting (half-life >24 hours) using pharmacokinetic data from the Ashton Manual.^18^ A full list and classification of benzodiazepines are included in the eMethods of the Supplement.

Using a similar approach, we ascertained use of CNS depressants as noted by the US Food and Drug Administration,^19^ including opioids, nonbenzodiazepine sedative hypnotics, muscle relaxants, and antipsychotics (see the eMethods in the Supplement for a full list). We then estimated the coprescribing rate of a benzodiazepine with a CNS depressant.

### Physician Specialty and Indication

We grouped visits into 4 categories based on the specialty of the physician with whom the visit was conducted: (1) primary care physicians (PCPs), defined by NAMCS as family medicine, internal medicine, geriatric medicine, and obstetrics and gynecology; (2) surgical specialties; (3) psychiatry; and (4) medical specialties. Using chief complaints (coded into NAMCS using the reason-for-visit [RFV] classification scheme)^20^ and *ICD-9-CM* diagnoses (eTable 1 in the Supplement), we separately assigned visits to 1 or more of the following categories of potential indications: (1) anxiety and depression; (2) back and chronic pain; (3) insomnia; (4) neurologic conditions (ie, headache, seizures, vertigo, and movement disorders); and (5) other. From 2003 through 2013, as many as 3 chief complaints and 3 diagnoses could be recorded per visit in the NAMCS. These increased to 5 in 2014 and 2015. To maintain consistency over time, we restricted our analyses to the first 3 positions of each.

For visits in which a benzodiazepine was recorded, the indication for that benzodiazepine was assumed to correspond to the RFV chief complaints and *ICD-9-CM* diagnoses that could reasonably be treated with a benzodiazepine. A benzodiazepine could be attributed to multiple indications. For example, if a benzodiazepine was noted in a visit that carried an RFV of anxiety and an *ICD-9-CM* code for insomnia, we attributed the benzodiazepine to both indications. In a sensitivity analysis, we verified the robustness of our attribution strategy by examining *ICD-9-CM* codes without RFV codes or excluding visits with multiple indications to which the benzodiazepine could be attributed.

### Statistical Analysis

Data were analyzed from July 1, 2017, through November 30, 2018. Unadjusted overall and stratified trends in benzodiazepine use were evaluated using the χ^2^ trend test. We then estimated trends using a logistic regression model that included a categorical indicator variable for year and adjusted for patient characteristics, including age, sex, race, insurance, region, and urban location.

We conducted 3 additional prespecified analyses. First, we examined trends in benzodiazepine coprescribing with opioids and other CNS depressants. Second, we separately evaluated trends in new vs continuing medications as well as short-acting vs long-acting benzodiazepines. Third, we used logistic regression pooled across the entire sample to evaluate patient-level factors independently associated with receiving a benzodiazepine prescription. We tested for effect modification using an interaction term between covariate and year to determine whether trends differed among categories. Aside from region, no effect modification occurred, so we report results from the full model including all years.

Per National Center for Health Statistics recommendations, to produce reliable estimates, all SEs were less than 30% of the estimate, and all sample sizes were greater than 30. Analyses were conducted using SAS (version 9.4; SAS Institute, Inc) and SAS-Callable SUDAAN (version 11.0; RTI International), which takes the complex survey design into account and produces national estimates. All statistical tests were 2 tailed with a level of significance set at *P* < .05. Because of the exploratory nature of the secondary outcomes, no corrections were made for multiple testing.^21^

## Results

We identified 20 884 visits in 2003 and 24 273 visits in 2015, representing an increase in the total number of ambulatory visits among adults in the United States from 737 million to 841 million during the study period (eTable 2 in the Supplement). In total, we studied 386 457 visits from 2003 to 2015, a mean (SD) of 29 727 (12 063) visits each year.

**Figure 1.**
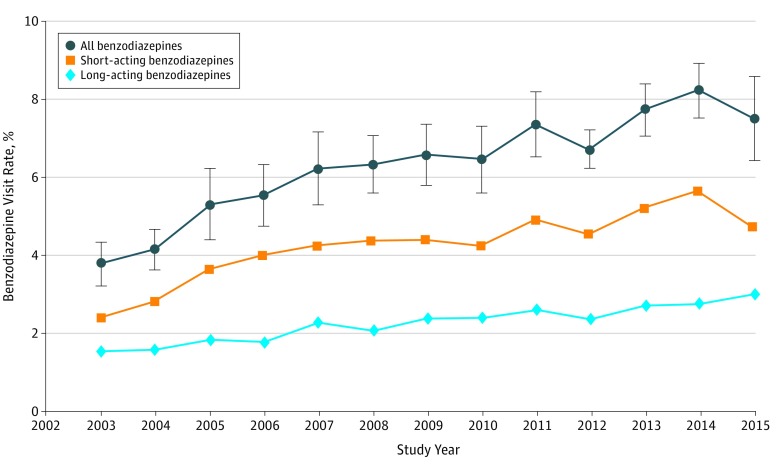
Benzodiazepine Visit Rate in the United States Error bars indicate 95% CI.

Among these visits, we identified 919 benzodiazepine visits in 2003 and 1672 benzodiazepine visits in 2015, nationally representing 27.6 million and 62.6 million visits, respectively (Table 1). The benzodiazepine visit rate increased from 3.8% (95% CI, 3.2%-4.4%) to 7.4% (95% CI, 6.4%-8.6%) of visits (*P* < .001), corresponding to an unadjusted increase of 95% from 2003 to 2015, or an adjusted odds ratio (OR) of 2.09 (95% CI, 1.67-2.62). The increase was similar for short-acting and long-acting benzodiazepines (Figure 1). When considered along with the increase in the total number of ambulatory visits, a 127% increase in the absolute number of benzodiazepine visits occurred. From 2005 to 2015, new prescriptions remained stable at 1.0% (changing from 0.9% [95% CI, 0.7%-1.2%] of visits in 2005 to 1.1% [95% CI, 0.7%-1.5%] in 2015; *P* = .64), but continuing prescriptions increased by 50% from 4.2% (95% CI, 3.4%-5.1%) to 6.4% (95% CI, 5.4%-7.6%) (*P* < .001).

**Table 1.** Benzodiazepine Visit Rate, Overall and by Visit Characteristics

Characteristic	No. (%) of Benzodiazepine Visits, 1 Million		Unadjusted Estimated Benzodiazepine Visit Rate, % (95% CI)			
	2003	2015	2003	2015	*P* Value^a^	Adjusted OR (95% CI)^b^
Overall	27.6	62.6	3.8 (3.2-4.4)	7.4 (6.4-8.6)	<.001	2.09 (1.67-2.62)
Age, y						
18-44	8.9 (32.2)	15.7 (25.1)	3.5 (2.8-4.5)	6.8 (5.4-8.7)	<.001	2.02 (1.41-2.88)
45-64	11.7 (42.4)	27.5 (43.9)	4.5 (3.8-5.4)	9.0 (7.5-10.9)	<.001	2.11 (1.61-2.77)
≥65	7.0 (25.4)	19.4 (31.0)	3.1 (2.5-3.8)	6.4 (5.3-7.8)	<.001	2.25 (1.69-3.00)
Sex						
Male	8.2 (29.7)	22.0 (35.1)	3.0 (2.4-3.6)	6.7 (5.4-8.3)	<.001	2.45 (1.78-3.38)
Female	19.4 (70.3)	40.6 (64.9)	4.2 (3.6-5.0)	7.9 (6.7-9.3)	<.001	1.95 (1.53-2.49)
Race						
White	24.9 (90.2)	52.9 (84.5)	3.9 (3.4-4.6)	8.1 (7.0-9.3)	<.001	2.09 (1.66-2.65)
Black	2.1 (7.6)	7.2 (11.5)	3.0 (2.1-4.4)	6.4 (4.0-10.0)	<.001	2.02 (1.12-3.64)
Other	0.6 (2.2)	2.5 (4.0)	1.8 (1.0-3.2)	3.5 (1.7-6.9)	.02	2.01 (0.82-4.95)
Insurance						
Private	13.3 (48.2)	26.1 (41.7)	3.4 (2.7-4.2)	6.9 (5.8-8.1)	<.001	2.17 (1.62-2.92)
Medicare	8.5 (30.8)	20.3 (32.4)	4.2 (3.5-5.1)	7.6 (6.3-9.1)	<.001	1.99 (1.52-2.61)
Medicaid	2.0 (7.2)	6.2 (1.0)	3.9 (2.8-5.5)	7.5 (5.0-11.1)	.03	1.98 (1.16-3.39)
Other^c^	3.7 (13.4)	10.0 (16.0)	4.4 (3.1-6.2)	9.1 (6.1-13.3)	<.001	2.29 (1.30-4.02)
Region						
Northeast	6.5 (23.6)	12.8 (20.4)	4.2 (2.9-6.1)	7.5 (4.9-11.1)	.001	1.83 (1.01-3.30)
Midwest	6.2 (22.5)	11.5 (18.4)	4.2 (2.9-6.1)	8.0 (6.4-9.8)	<.001	1.94 (1.24-3.03)
South	10.8 (39.1)	19.8 (31.6)	4.0 (3.3-4.8)	6.5 (5.2-8.2)	<.001	1.70 (1.24-2.34)
West	4.1 (14.9)	18.5 (29.6)	2.6 (1.8-3.6)	8.4 (6.3-11.0)	<.001	3.61 (2.25-5.79)
Location						
Urban	22.9 (83.0)	59.0 (94.2)	3.5 (3.0-4.2)	7.5 (6.5-8.7)	<.001	2.23 (1.75-2.85)
Rural	4.7 (17.0)	3.6 (5.8)	5.3 (4.0-7.1)	6.4 (4.1-10.1)	<.001	1.20 (0.67-2.13)

Abbreviation: OR, odds ratio.

^a^ Calculated using χ^2^ trend test.

^b^ Adjusted for age, sex, race, insurance, region, and location.

^c^ Includes uninsured, worker’s compensation, self-pay, charity, or unknown.

### Use by Physician Specialty and Indication

**Figure 2.**
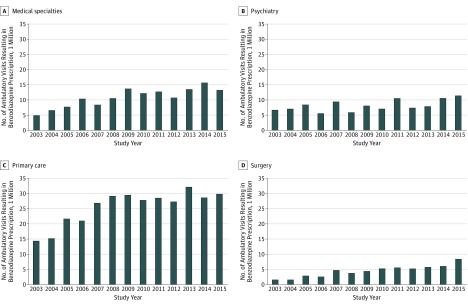
Benzodiazepine Visits by Specialty

After stratifying visits by specialty, visits to PCPs accounted for about half of all benzodiazepine visits (52.3% [95% CI, 50.0%-54.6%]; changing from 51.4% [95% CI, 43.1%-61.2%] in 2003 to 47.7% [95% CI, 38.0%-57.1%] in 2015), followed by visits to medical specialists (22.0% [95% CI, 20.1%-24.1%]; changing from 17.4% [95% CI, 12.6%-24.0%] to 20.9% [95% CI, 14.6%-29.2%]), psychiatrists (16.6% [95% CI, 15.2%-18.2%]; changing from 24.3% [95% CI, 17.8%-32.2%] to 18.8% [95% CI, 12.2%-26.0%]), and surgeons (9.1% [95% CI, 8.2%-9.9%]; changing from 5.8% [95% CI, 3.5%-10.0%] to 13.1% [95% CI, 9.9%-17.9%]) (Figure 2). The unadjusted benzodiazepine visit rate did not change among visits to psychiatrists (29.6% [95% CI, 23.3%-36.7%] to 30.2% [95% CI, 25.6%-35.2%]; *P* = .90), but increased among visits to PCPs (3.6% [95% CI, 2.9%-4.4%] to 7.5% [95% CI, 6.0%-9.5%]; *P* < .001), surgeons (1.0% [95% CI, 0.6%-1.6%] to 4.3% [95% CI, 3.5%-5.5%]; *P* < .001), and medical specialists (3.3% [95% CI, 2.4%-4.5%] to 6.0% [95% CI, 4.5%-7.9%]; *P* < .001) (for year-by-year estimates, see eTable 3 in the Supplement).

After stratifying by indication (Table 2), the unadjusted benzodiazepine visit rate increased for anxiety and depression (26.6% [95% CI, 22.6%-31.0%] to 33.5% [95% CI, 28.8%-38.6%], *P* = .003) and neurologic conditions (6.8% [95% CI, 4.8%-9.5%] to 8.7% [95% CI, 6.2%-12.1%]; *P* < .001), but more so for back and chronic pain (3.6% [95% CI, 2.6%-4.9%] to 8.5% [95% CI, 6.0%-11.9%]; *P* < .001) and instances in which we were unable to attribute the benzodiazepine to a particular indication (1.8% [95% CI, 1.4%-2.2%] to 4.4% [95% CI, 3.7%-5.2%]; *P* < .001). Use did not change for insomnia (26.9% [95% CI, 19.3%-36.0%] to 25.6% [95% CI, 15.3%-39.6%]; *P* = .72). Results from adjusted and sensitivity analyses were similar.

**Table 2.** Benzodiazepine Visit Rate by Indication

Indication^a^	No. of Benzodiazepine Visits, 1 Million		Unadjusted Estimated Benzodiazepine Visit Rate, % (95% CI)			
	2003	2015	2003	2015	*P* Value^b^	Adjusted OR (95% CI)^c^
Anxiety and depression	12.8	23.7	26.6 (22.6-31.0)	33.5 (28.8-38.6)	.003	1.43 (1.05-1.95)
Back and chronic pain	4.9	15.1	3.6 (2.6-4.9)	8.5 (6.0-11.9)	<.001	2.65 (1.65-4.26)
Insomnia	2.1	3.4	26.9 (19.3-36.0)	25.6 (15.3-39.6)	.72	0.94 (0.46-1.92)
Neurologic^d^	3.3	5.0	6.8 (4.8-9.5)	8.7 (6.2-12.1)	<.001	1.37 (0.85-2.22)
Other	9.1	24.5	1.8 (1.4-2.2)	4.4 (3.7-5.2)	<.001	2.50 (1.90-3.29)

Abbreviation: OR, odds ratio.

^a^ A visit can be ascribed to multiple diagnoses.

^b^ Calculated using χ^2^ trend test.

^c^ Adjusted for age, sex, race, insurance, region, and location.

^d^ Includes headache, seizures, vertigo, and movement disorders.

### Coprescribing and Predictors Associated With Use

**Figure 3.**
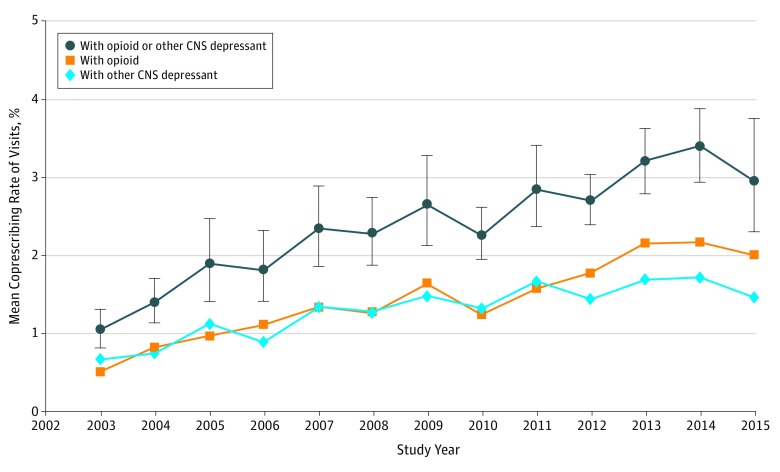
Coprescribing Rate for Benzodiazepines With Opioids and Other Central Nervous System (CNS) Depressants Error bars indicate 95% CI.

Commensurate with the increase in benzodiazepine use, the rate at which benzodiazepine and opioid prescriptions were noted in a single visit quadrupled from 0.5% (95% CI, 0.3%-0.7%) to 2.0% (95% CI, 1.4%-2.7%) (*P* < .001) (Figure 3). In 2015, benzodiazepines were coprescribed in 19.2% (95% CI, 16.3%-21.0%) of visits in which there was also an opioid; similarly, opioids were coprescribed in 26.4% (95% CI, 20.8%-33.7%) of visits in which there was also a benzodiazepine. Between 2003 and 2015, the coprescribing rate of benzodiazepines with other CNS depressants (nonbenzodiazepine sedative hypnotics, muscle relaxants, and antipsychotics) more than doubled from 0.7% (95% CI, 0.5%-0.9%) to 1.5% (95% CI, 1.1%-1.9%) (*P* < .001). Overall, the coprescribing rate with opioids or other CNS depressants increased from 1.0% (95% CI, 0.8%-1.3%) to 2.9% (95% CI, 2.3%-3.8%) of visits (*P* < .001).

In a multivariable logistic regression model examining predictors associated with use (eTable 4 in the Supplement), we found that women (OR, 1.31 [95% CI, 1.24-1.38]), middle-aged adults (OR for ages 45-64 years, 1.40 [95% CI, 1.33-1.48]), and those with public insurance (OR for Medicare, 1.81 [95% CI, 1.69-1.95]; OR for Medicaid, 1.54 [95% CI, 1.38-1.71]) were more likely to be prescribed benzodiazepines. Nonwhite patients (OR for black patients, 0.63 [95% CI, 0.56-0.70]; OR for other races, 0.52 [95% CI, 0.44-0.62]) were less likely to be prescribed benzodiazepines.

## Discussion

Using nationally representative data, we surveyed the landscape of outpatient benzodiazepine use and found that the rate for benzodiazepine visits doubled from 2003 to 2015. Use among psychiatrists was stable, but increased among all other types of physicians, including PCPs, medical specialists, and surgeons. By indication, use was stable for visits related to insomnia and increased by only about one-quarter for visits related to anxiety or neurologic conditions; in contrast, it more than doubled for back and chronic pain as well for other conditions for which we could not identify a specific indication. In addition, benzodiazepines are increasingly prescribed with other sedating medications.

The rising rate of overdose mortality involving benzodiazepines is likely multifactorial, but our results provide insights into potential underlying causes. A previous study^22^ showed that the benzodiazepine visit rate increased from 2.6% in 1993 to 4.4% in 2010. We extended these results by looking at more recent data through 2015 (showing that the rate continued to increase to 7.4%), stratifying visits by specialty and by indication and examining coprescribing in greater depth. The increase in the number of benzodiazepine visits likely reflects not only a growing number of unique individuals receiving benzodiazepines, but also an increase in those who are receiving benzodiazepines on a long-term basis. Other studies using pharmaceutical claims data and the National Health and Nutrition Examination Survey support the conclusion that long-term benzodiazepine use may be a larger driver of the increased use of this class of medications.^23,24^ This finding is of even greater concern because little evidence supports the use of benzodiazepines past 8 or 10 weeks, as suggested by US Food and Drug Administration labeling and several disease-specific clinical guidelines.^25,26,27,28,29^

Examination of trends in use by specialty and indication revealed important patterns with respect to how benzodiazepines are used and prescribed. First, although use has increased among all specialties except psychiatry, primary care may be the source of the plurality, if not most, of benzodiazepine prescriptions. Second, although a modest increase occurred in use for anxiety and unchanged use for insomnia, we found that benzodiazepine use for back and chronic pain as well as undefined indications increased by a much larger degree. One possibility that might explain these trends is the greater availability and effectiveness of other classes of medications for anxiety and insomnia, while the options for pain remain more limited. Our understanding of the opioid epidemic may also be instructive. In particular, these trends could reflect an underappreciation of the risks associated with benzodiazepines and an overappreciation of the benefits, given their rapid therapeutic effects,^30^ marketing techniques used by the pharmaceutical industry,^31^ greater frequency with which anxiety or other “diseases of despair” are manifesting themselves in presentations to primary care,^32^ and poor availability of or access to pharmacologic and nonpharmacologic alternatives. Moreover, as opioids lose favor among prescribers, we must remain cognizant that this might lead to increased use of other potentially dangerous drugs such as benzodiazepines, especially because evidence for their use in conditions such as back pain is limited.^33^ Ultimately, any efforts to address or curb benzodiazepine use should address use within primary care, for instance through the development of benzodiazepine-specific guidelines or better tracking via prescription monitoring programs.

Although our study does not separate appropriate vs inappropriate use, the risks of benzodiazepines more likely outweigh the benefits when they are used in combination with other CNS depressants. The Centers for Disease Control and Prevention and US Food and Drug Administration issued warnings in 2016 to make prescribers and patients aware of these risks.^19,34^ Previous studies found that coprescribing with opioids is common.^13,35,36,37^ Those studies, however, did not examine coprescribing with other sedating medications and focused on overlapping prescriptions periods, which could be the result of prescriptions given to a patient at different times or by different prescribers. Our study showed that, within a visit, coprescribing has increased for not only benzodiazepines and opioids, but also benzodiazepines and nonbenzodiazepine sedative hypnotics, muscle relaxants, and antipsychotics.

### Limitations

Our analysis has several limitations. First, minor changes in survey design and data collection procedures for the NAMCS occur from year to year. We sought to minimize some of these effects in the design of our study, for example, by consistently examining only the first 8 medications listed in the survey. However, secular trends, such as widespread adoption of the electronic medical record system, could explain some of the trends we observed. Second, the NAMCS lacks detail on dosage, dosing frequency, refills, and long-term use of a prescription. The increasing availability of prescription claims with such detail presents an opportunity for further investigation. Third, the NAMCS is representative of visits, not patients. We are therefore unable to examine certain outcomes of interest, such as subsequent refills or hospitalizations for overdose. Fourth, our ability to attribute a prescription to an indication was imperfect. We assumed that the chief complaints or diagnoses coded during a visit when a benzodiazepine was noted corresponded to the indications of that benzodiazepine. In our classification scheme, the other category included nonspecific diagnoses, such as cancer or general medical examination. Initiatives to incorporate indications-based prescribing into electronic medical records are in their infancy, but would begin to provide better data for future research. Fifth, our results are generalizable to nonfederal office-based physician practices, the population that NAMCS targets for its nationally representative sample, and do not necessarily include hospital-based outpatient clinics. Nonetheless, we would expect similar trends in these settings.

## Conclusions

Surprisingly few guidelines exist for a medication that is prescribed by so many different types of physicians and for so many different indications. Benzodiazepines can be useful and effective medications when prescribed selectively in appropriate patients for short-term use. However, our results reveal that use of benzodiazepines in ambulatory care has increased substantially, including coprescribing with other sedating medications. Primary care physicians accounted for the most benzodiazepines visits, and benzodiazepine use has risen substantially for indications other than anxiety and insomnia. As we have seen with the opioid epidemic and in light of increasing death rates related to benzodiazepine overdose, addressing prescribing patterns may help curb the growing use of benzodiazepines.

## Notes

Supplement. **eMethods.** List of Benzodiazepines, Opioids, Nonbenzodiazepine Sedative Hypnotics (and Other Sleep Drugs), Muscle Relaxants, and Antipsychotics **eTable 1.** RFV and *ICD-9-CM* Codes for Indication Categories **eTable 2.** Characteristics of All Visits, 2003-2015 **eTable 3.** Benzodiazepine Prescribing Rate by Year, by Specialty and Indication, 2003-2015 **eTable 4.** Predictors Associated With Use of Benzodiazepines
