# Incorrect Column Headings in 2 Tables

**DOI:** 10.1001/jamanetworkopen.2019.1203

**Published:** 2019-03-01

In the Original Investigation titled “Patterns in Outpatient Benzodiazepine Prescribing in the United States,”^1^ published January 25, 2019, the headings for 2 columns were incorrect in Tables 1 and 2. In both tables, the sample sizes should not have appeared in the headings for the first 2 data columns, and the word “unweighted” should not have appeared in the spanning heading above those columns because the data are weighted. This article has been corrected.^1^
