# Supplementary material for: Patterns in Outpatient Benzodiazepine Prescribing in the United States
Source: JAMA Netw Open. 2019 Jan 25;2(1):e187399. doi: 10.1001/jamanetworkopen.2018.7399 (PMC6484578; doi:10.1001/jamanetworkopen.2018.7399)
Supplement: Supplement. — eMethods. List of Benzodiazepines, Opioids, Nonbenzodiazepine Sedative Hypnotics (and Other Sleep Drugs), Muscle Relaxants, and Antipsychotics eTable 1. RFV and ICD-9-CM Codes for Indication Categories eTable 2. Characteristics of All Visits, 2003-2015 eTable 3. Benzodiazepine Prescribing Rate by Year, by Specialty and Indication, 2003-2015 eTable 4. Predictors Associated With Use of Benzodiazepines [file jamanetwopen-2-e187399-s001.pdf]

Supplementary Online Content

Agarwal SD, Landon BE. Patterns in outpatient benzodiazepine prescribing in the United States. *JAMA Netw Open*. 2019;2(1):e187399. doi:10.1001/jamanetworkopen.2018.7399

eMethods. List of Benzodiazepines, Opioids, Nonbenzodiazepine Sedative Hypnotics (and Other Sleep Drugs), Muscle Relaxants, and Antipsychotics

eTable 1. RFV and *ICD-9-CM* Codes for Indication Categories

eTable 2. Characteristics of All Visits, 2003-2015

eTable 3. Benzodiazepine Prescribing Rate by Year, by Specialty and Indication, 2003-2015

eTable 4. Predictors Associated With Use of Benzodiazepines

This supplementary material has been provided by the authors to give readers additional information about their work.

© 2019 Agarwal SD et al. *JAMA Network Open.*

eMethods. List of Benzodiazepines, Opioids, Nonbenzodiazepine Sedative Hypnotics (and Other Sleep Drugs), Muscle Relaxants, and Antipsychotics

Full list of benzodiazepines (generic and brand-name)

Starred (*) entries indicate benzodiazepines that were categorized as short-acting. All others were categorized as long-acting.

- Chlordiazepoxide, Chlordiazepoxide HCl with clidinium bromid, Librix, Librium
- *Alprazolam, *Xanax, *Xanax XR, Niravam
- *Oxazepam, *Serax
- Diazepam, Valium, Diastat, Diastat Acudial
- Clonazepam, Klonopin
- *Estazolam, *Prosom
- Flurazepam, Dalmane
- *Triazolam, *Halcion
- Clorazepate, Clorazepate dipotassium, Tranxene T, Tranxene
- *Lorazepam, *Ativan
- *Temazepam, *Restoril
- Clobazam, Onfi
- *Midazolam, *Midazolam HCl, *Versed

© 2019 Agarwal SD et al. *JAMA Network Open.*

Full list of opioids, non-benzodiazepine sedative-hypnotics (and other sleep drugs), muscle relaxants, and antipsychotics

Opioids

- Buprenorphine, Buprenorphine-Naloxone, Buprenex, Butrans, Subutex
- Butorphanol Tartrate, Stadol
- Codeine, Acetaminophen with Codeine, APAP with Codeine, Codeine Phosphate, Codeine Sulfate, Butalbital-ASA-Caffeine-Codeine, Fiorinal with Codeine, Guaifenesin with Codeine, Phenergan Vc Expectorant with Codeine, Phenergan with Codeine, Promethazine Vc with Codeine, Promethazine with Codeine, Tylenol with Codeine, Robitussin with Codeine, Fioricet/Codeine
- Fentanyl, Fentanyl Citrate, Duragesic, Fentora, Subsys, Sublimaze
- Hydrocodone, Homatropine-Hydrocodone, Guaifenesin-Hydrocodone, Chlorpheniramine/Hydrocodone, Hydrocodone/Ibuprofen, Hydrocodone with Acetaminophen, Hydrocodone Compound, Acetaminophen-Hydrocodone, Lortab Elixir, Lortab, Norco, Vicodin, Vicodin ES, Vicodin HP
- Hydromorphone, Dilaudid, Exalgo
- Meperidine, Meperidine HCl, Demerol, Meperitab
- Methadone, Dolophine
- Morphine, Morphine ER, Morphine Sulfate, MS Contin, Kadian, Roxanol, Avinza, Duramorph
- Nalbuphine HCl, Nubain
- Nucynta

© 2019 Agarwal SD et al. *JAMA Network Open.*

- Oxycodone, Acetaminophen W/Oxycodone, Oxycodone ER, Oxycodone CR, Oxycodone HCl, Oxycodone HCl & Acetaminophen, Oxycodone with APAP, Oxycodone with ASA, Oxycontin, Roxicodone, OxyIR, Oxyfast, Percocet, Percocet-5, Percocet 7.5, Percocet 10
- Oxymorphone, Opana, Opana ER
- Propoxyphene, Propoxyphene HCl with Acetaminophen, Darvon, Darvon Compound
- Talwin Nx
- Tramadol, Tramadol HCl, Acetaminophen-Tramadol, Ultram ER, Ultram

Non-benzodiazepine sedative-hypnotics (and other sleep drugs)

- Eszopiclone, Lunesta
- Zolpidem, Zolpidem Tartrate, Ambien CR, Ambien, Edluar, Intermezzo
- Zaleplon, Sonata
- Ramelteon, Rozerem
- Belsomra

Muscle relaxants

- Baclofen, Lioresal
- Carisoprodol, Soma
- Chlorzoxazone
- Cyclobenzaprine HCl, Flexeril, Amrix
- Dantrolene
- Metaxalone, Skelaxin
- Methocarbamol, Robaxin-750, Robaxin

© 2019 Agarwal SD et al. *JAMA Network Open.*

- Orphenadrine
- Tizanidine , Zanaflex

Antipsychotics

- Aripiprazole, Abilify
- Asenapine, Saphris
- Chlorpromazine
- Clozapine, Clozaril, Fazaclo
- Fluphenazine, Fluphenazine Decanoate
- Haloperidol, Haloperidol Deconate, Haloperidol Lactate, Haldol Decanoate, Haldol
- Fanapt
- Loxapine
- Lurasidone, Latuda
- Olanzapine, Olanzapine/Fluoxetine, Zyprexa, Zyprexa Zydis
- Paliperidone, Invega, Invega Sustenna
- Perphenazine, Perphenazine with Amitriptyline, Amitriptyline HCl with Perphenazine
- Quetiapine Fumarate, Seroquel XR, Seroquel
- Risperidone, Risperdal
- Thioridazine
- Thiothixene , Navane
- Trifluoperazine
- Ziprasidone HCl, Geodon

© 2019 Agarwal SD et al. *JAMA Network Open.*

eTable 1. RFV and *ICD-9-CM* Codes for Indication Categories

Indication	RFV and ICD-9-CM Codes
Anxiety and depression	ICD-9-CM:<br>290.13, 290.21, 290.43, 296.2-, 296.20, 296.21, 296.22, 296.23, 296.24, 296.25, 296.26, 296.3-, 296.30, 296.31, 296.32, 296.33, 296.34, 296.35, 296.36, 296.9-, 296.90, 296.99, 300.00, 300.01, 300.02, 300.09, 300.20, 300.21, 300.22, 300.23, 300.29, 300.3-, 300.4-, 308.0-, 308.3-, 308.4-, 308.9-, 309.0-, 309.1-, 309.24, 309.28, 309.29, 309.81, 311.-, V11.2, V79.0<br><br>RFV:<br>1100.0, 1105.0, 1110.0, 2310.0
Back and chronic pain	ICD-9-CM:<br>307.80, 338, 354, 355, 356, 357, 370.89, 719.4, 720.1-, 721, 722, 723, 724, 728.85, 729.8, 729.1-, 729.2-, 729.5-, 729.9-, 729.90, 729.99, 737, 739.0-, 739.1-, 739.2-, 739.3-, 739.4-, 739.5-, 739.6-, 739.7-, 739.8-, 780.96, 840, 841, 842, 843, 844, 845, 846, 847, 848<br><br>RFV:<br>1055.0, 1060.0, 1060.1, 1060.2, 1060.3, 1900.0, 1900.1, 1900.2, 1900.3, 1905.0, 1905.1, 1905.2, 1905.3, 1910.0, 1910.1, 1910.2, 1910.3, 1915.0, 1915.1, 1915.2, 1915.3, 1920.0, 1920.1, 1920.2, 1920.3, 1925.0, 1925.1, 1925.2, 1925.3, 1930.0, 1930.1, 1930.2, 1930.3, 1935.0, 1935.1, 1935.2, 1935.3, 1940.0, 1940.1, 1940.2, 1940.3, 1945.0, 1945.1, 1945.2, 1945.3, 1950.0, 1950.1, 1950.2, 1950.3, 1955.0, 1955.1, 1955.2, 1955.3, 1960.0, 1960.1, 1960.2, 1960.3, 1965.0, 1965.1, 1965.2, 1965.3, 1970.0, 1970.1, 1970.2, 1970.3, 1980.0, 2900.0, 2905.0, 2910.0
Insomnia	ICD-9-CM:<br>307.4-, 307.40, 307.41, 307.42, 307.45, 307.46, 307.47, 307.48, 307.49, 327.0-, 327.00, 327.01, 327.02, 327.09, 780.5-, 780.50, 780.51, 780.52, 780.55, 780.56, 780.59, V69.4-<br><br>RFV:<br>1135.0, 1135.1
Neurologic conditions	ICD-9-CM:<br>307.81, 327.52, 332, 333, 339, 340, 345, 346, 386, 438.85, 780.3, 780.4-, 781.0-, 784<br><br>RFV:<br>1200.0, 1205.0, 1207.0, 1210.0, 1220.0, 1220.1, 1220.2, 1220.3, 1220.4, 1225.0, 1410.1, 2365.0

© 2019 Agarwal SD et al. *JAMA Network Open.*

eTable 2. Characteristics of All Visits, 2003-2015

Characteristic	Number of Ambulatory Visits (%*)		
	**2003** (unweighted N = 20,884)	**2015** (unweighted N = 24,273)	**Total** (unweighted N = 386,457)
U.S. weighted population of visits	736,631,072	841,252,183	10,126,576,829
Age			
18-44	6,639 (34.2)	6,428 (27.4)	116,099 (30.9)
45-64	7,359 (34.9)	8,311 (36.3)	140,361 (36.2)
≥65	6,886 (30.9)	9,534 (36.3)	129,997 (32.9)
Sex			
Male	8,724 (37.9)	10,386 (39.1)	157,463 (39.1)
Female	12,160 (62.1)	13,887 (60.9)	228,994 (60.9)
Race			
White	18,387 (85.8)	20,942 (78.0)	331,350 (84.5)
Black	1,756 (9.4)	2,299 (13.3)	37,502 (10.4)
Other	741 (4.8)	1,032 (8.7)	17,605 (5.1)
Insurance			
Private	10,926 (54.1)	11,362 (45.2)	185,335 (50.5)
Medicare	6,157 (27.5)	8,300 (31.9)	117,258 (29.5)
Medicaid	1,320 (6.9)	2,149 (9.8)	30,114 (7.5)
Other	2,481 (11.5)	2,462 (13.1)	53,750 (12.5)
Region			
Northeast	4,451 (21.0)	4,558 (20.4)	67,836 (19.7)
Midwest	4,417 (20.2)	7,304 (17.2)	95,716 (20.2)
South	7,376 (37.0)	6,685 (36.1)	131,645 (38.0)
West	4,640 (21.8)	5,726 (26.3)	91,260 (22.1)
Location			
Urban	18,489 (88.0)	22,458 (93.4)	341,250 (88.9)
Rural	2,395 (12.0)	1,815 (6.6)	45,207 (11.1)

* Percentages are weighted to provide population estimates.

© 2019 Agarwal SD et al. *JAMA Network Open.*

eTable 3. Benzodiazepine Prescribing Rate by Year, by Specialty and Indication, 2003-2015

	Ambulatory Visits Resulting in Benzodiazepine Prescription, %												
**Characteristic**	**2003**	**2004**	**2005**	**2006**	**2007**	**2008**	**2009**	**2010**	**2011**	**2012**	**2013**	**2014**	**2015**
All	3.8	4.1	5.2	5.5	6.2	6.3	6.5	6.4	7.3	6.7	7.7	8.2	7.4
Specialty													
Primary care	3.6	3.9	5.2	5.7	6.5	6.8	6.7	6.9	7.1	7.5	8.6	8.2	7.5
Medical specialties	3.3	4.2	5.2	6.3	4.8	7.0	6.5	6.2	6.8	5.4	6.7	8.0	6.0
Surgery	1.0	0.9	1.6	1.6	2.6	2.0	2.5	2.7	3.5	3.1	3.5	3.8	4.4
Psychiatry	29.6	26.8	33.6	26.8	31.1	29.6	29.4	32.1	32.9	28.8	25.7	29.1	30.2
Indication													
Anxiety & depression	26.6	23.9	29.2	28.0	31.7	34.8	30.6	32.4	34.6	30.0	30.5	32.5	33.5
Back & chronic pain	3.6	4.7	5.1	6.0	7.7	7.0	7.6	6.4	9.0	9.0	10.4	9.5	8.5
Insomnia	26.9	21.2	18.9	23.4	21.9	27.8	27.4	21.7	22.4	25.6	22.0	24.0	25.6
Neurologic	6.8	6.3	7.2	7.1	10.0	8.9	10.2	8.4	10.0	9.7	11.1	11.7	8.7
Other	1.8	2.2	3.4	3.7	3.5	4.0	4.1	4.4	4.6	4.0	4.6	5.0	4.4

© 2019 Agarwal SD et al. *JAMA Network Open.*

eTable 4. Predictors Associated With Use of Benzodiazepines

Predictor	Adjusted OR [95% CI] [a]
Age	
18-44	1.00
45-64	1.40 [1.33, 1.48]
≥65	0.79 [0.73, 0.86]
Sex	
Male	1.00
Female	1.31 [1.24, 1.38]
Race	
White	1.00
Black	0.63 [0.56, 0.70]
Other	0.52 [0.44, 0.62]
Insurance	
Private	1.00
Medicare	1.81 [1.69, 1.95]
Medicaid	1.54 [1.38, 1.71]
Other/ uninsured	1.36 [1.23, 1.49]
Region	
Northeast	1.00
Midwest	1.04 [0.91, 1.18]
South	1.08 [0.93, 1.24]
West	0.98 [0.86, 1.11]
Location	
Urban	1.00
Rural	1.07 [0.92, 1.25]

[a] Adjusted for year, age, sex, race, insurance, region, and location.

© 2019 Agarwal SD et al. *JAMA Network Open.*
